# Do You Remember Mitochondria?

**DOI:** 10.3389/fphys.2020.00271

**Published:** 2020-03-27

**Authors:** Flavia Messina, Francesco Cecconi, Carlo Rodolfo

**Affiliations:** ^1^Department of Biology, University of Rome Tor Vergata, Rome, Italy; ^2^Department of Paediatric Haematology, Oncology, and Cell and Gene Therapy, IRCCS Bambino Gesù Children’s Hospital, Rome, Italy; ^3^Unit of Cell Stress and Survival, Danish Cancer Society Research Center, Copenhagen, Denmark

**Keywords:** mitochondria, neurodegenerative diseases, mitophagy and mitochondrial dynamics, reactive oxygen species, aging

## Abstract

Dementia is one among the consequences of aging, and amnesia is often one of the most common symptoms. The lack of memory, as a consequence of both “healthy” aging or neurodegenerative conditions, such as in Alzheimer’s disease, has a dramatic impact on the patient’s lifestyle. In fact, the inability to recall information made by a previous experience could not only alter the interaction with the environment, but also lead to a loss of identity. Mitochondria are key regulators of brain’s activity; thanks to their “dynamic organelles” nature they constantly rearrange in the cell body and move along axons and dendrites, changing in dimension, shape, and location, accordingly to the cell’s energy requirements. Indeed, the energy they can provide is essential to maintain synaptic plasticity and to ensure transmission through presynaptic terminals and postsynaptic spines. Stressful conditions, like the ones found in neurodegenerative diseases, seriously impair mitochondria bioenergetic, leading to both loss of proper neuronal interaction and of neuron themselves. Here, we highlighted the current knowledge about the role of mitochondria and mitochondrial dynamics in relation to neurodegenerative disorders linked to aging. Furthermore, we discuss the obstacles as well as the future perspectives aimed to enlarge our knowledge about mitochondria as target for new therapeutic strategies to slow down aging and neurodegenerative disease’s symptoms.

## Introduction

Aging is characterized by a time-dependent impairment of physiological functions due to an accumulation of changes, at both cellular and molecular level, which could result in pathological conditions such as cancer, neurodegeneration, obesity, and cardiovascular disorders (López-Otín et al., 2013).

Among other organs, the brain is most sensitive to the aging process, since neurodegenerative conditions leading to dementia, Alzheimer’s, Parkinson’s, and Huntington’s disease exponentially increase with age progression (Mattson and Arumugam, 2018). One of the main causes of brain’s sensitivity relies on its high energy requests. In fact, in order to perform neuro-electrical transmission, neurons require a high rate of ATP, as to maintain ionic gradient, preserve Ca^2+^ equilibrium, and regulate the synaptic vesicle recycling. These activities result in the consumption of up to 20% of the total ATP produced in the whole body. Glucose metabolism is the main energy source in the brain, and as a consequence neurons deeply rely on mitochondrial oxidative phosphorylation (OXPHOS) system to produce ATP. Furthermore, the neuronal network depends upon synaptic plasticity, which is severely affected by lack of energy, resulting in impaired information transmission with consequent loss of memory and learning. Thus, it is not surprising that a disturbance of normal mitochondria physiology/functionality could lead to a decrease in neurons activity, and eventually cell death and neurodegeneration (Sun et al., 2016; Grimm and Eckert, 2017).

Nowadays, the involvement of mitochondrial dysfunction in the onset and development of neurodegenerative diseases has been extensively investigated (Beal, 2005; Cabral-Costa and Kowaltowski, 2019; Panchal and Tiwari, 2019). In Alzheimer’s disease (AD) a reduced electron transport activity has been associated with increased production of reactive oxygen species (ROS). In addition, amyloid beta and Tau accumulation at mitochondria results in organelle’s damage as well as in the impairment of mitochondrial trafficking and removal. Parkinson’s disease (PD) is characterized by an impairment in the mitochondrial respiratory Complex I activity, leading to altered Ca^2+^ homeostasis and ROS generation, by the presence of mtDNA mutations, and by mutations in many of the genes involved in the removal of the damaged organelles through the mitophagy process, such as Parkin, PINK1, LRKK2, DJ-1. Huntington’s disease (HD) is characterized by reduced Complex II activity, impaired Ca^2+^ uptake capacity, oxidative imbalance, and reduced mitochondria turnover (Beal, 2005; Cabral-Costa and Kowaltowski, 2019; Panchal and Tiwari, 2019). In addition to the disease-specific hallmarks, a commune feature of these disorders is the impairment of the mitochondrial quality control system, mainly due to a failure in the mitophagy process (Rodolfo et al., 2017). Nevertheless, at present there are not enough clues about the possible therapeutic advantages and/or applicability of mitophagy manipulation.

Another common feature of neurodegenerative conditions in the elders relies not only on the loss of neuronal plasticity but also of the neurons involved in the long term memory’s storage, such as Engram cells (Roy et al., 2016). The role played by mitochondria and mitochondrial dynamics in the process of long term memory engravement and recall is not at all clear, but quite surely they should play an important one. Moreover, as mitochondria and mitochondrial dynamics, have been shown to be important in the establishment of cellular memory in muscle stem cells (Cheikhi et al., 2019), should we imagine a similar role for mitochondria in the neuron? Could we imagine to, somehow, interfere with these processes in order to sustain, recall, or restore memory in the elders? How the knowledge acquired during the last decade, about some of the main cellular processes involved in the maintenance of organelle’s quality and homeostasis (Barbosa et al., 2019), would open a new scenario in the therapeutic approaches to diseases linked to aging?

## Ros and Mitochondria in Aging Progression

Aging, seen as a phenomena of growth, decline and death, is the “natural” consequence of an impairment of cellular functions, in particular of the decline in mitochondrial function and metabolic rate. In the “free radical theory of aging,” Harman postulated that aging is the consequence of the damage to cells and tissues, due to a progressive increase in the levels of free radicals (Harman, 1956). ROS are by-products of normal cellular activities, and mitochondria are one of the most important source of ROS (mtROS). ATP production by the electron transport chain, in particular complex I and III, generate superoxide radical (O_2_•−) and molecular oxygen (O_2_), which is finally reduced to water (Grimm and Eckert, 2017). In physiological conditions, ROS participate in cell signaling and are involved in processes, such as immune response, inflammation, synaptic plasticity, memory, and learning (Kishida and Klann, 2007; Snezhkina et al., 2019). The unbalanced increase in ROS levels results in oxidative stress, which could perturb cell’s homeostasis, structures, and functions, and in turn leading to pathological conditions, such as AD, PD, and cancer (Snezhkina et al., 2019). Cells and mitochondria protect themselves from ROS damage through the activity of a number of enzymatic defense systems, such as: glutathione peroxidases (GPXs), thioredoxin peroxidases (TRXPs), superoxide dismutases (SODs), peroxiredoxins (PRDXs), glutathione (GSH), thioredoxin 2 (TRX2), glutaredoxin 2 (GRX2), cytochrome c oxidase (complex IV); as well as antioxidants molecules, such as coenzyme Q, ascorbic acid, tocopherol, vitamin E, and carotene. Nevertheless, synaptic transmission, maintenance of membrane potential, functionality of ion channels, and synaptic plasticity in the brain, depends upon the energy provided by mitochondria, thus rendering this organ more sensitive to ROS accumulation and damage (Terman et al., 2010). In fact, during aging as well as in neurodegenerative diseases, the brain shows an increase in ROS production, coupled with a lowering of the antioxidant mechanisms, suggesting a direct correlation between oxidative stress and neuronal death (Venkateshappa et al., 2012). Maintenance of mitochondrial functionality is thus crucial not only for neuronal survival but could also guarantee the activities linked to memory engravement and recall.

**FIGURE 1:**
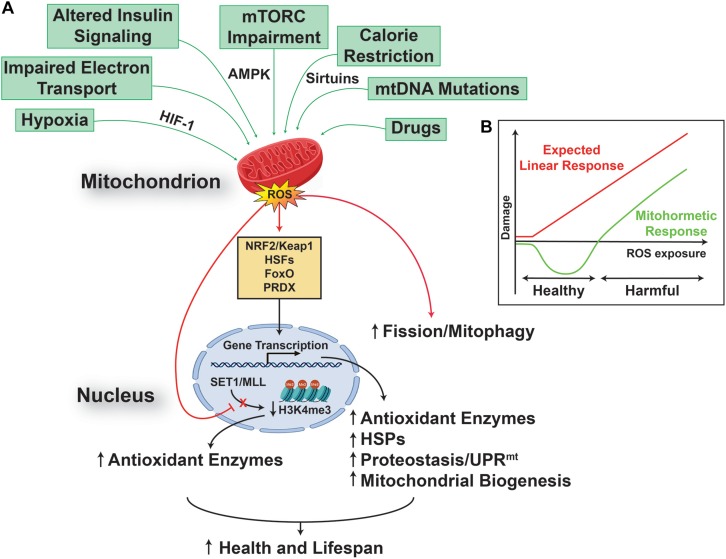
Reactive oxygen species promote health and lifespan. **(A)** Multiple mitochondria converging stresses results in an increased production of mitochondrial reactive oxygen species (mtROS), which are able to activate a transcriptional retrograde response, between the organelle and the nucleus, linking mtROS to nuclear events. The result of this pathway is an increased cell stress resistance that can extend health and lifespan. **(B)** This effect relies on a mitochondrial process called mitohormesis, in which mtROS engages a non-linear response (green), instead of the linear response (red) postulated by the classical free radical theory of aging. The mitohormetic response (green) is characterized by health span-promoting effects at low mtROS doses, while cellular and systemic damage are the consequence of higher mtROS doses.

On the other side, in the last decade, the free radical theory of aging has been challenged by numerous observations reporting the beneficial effect of mtROS on health and lifespan, in different animal models, ranging from worms to mammals (Lee et al., 2003; Dell’agnello et al., 2007; Rea et al., 2007; Copeland et al., 2009). In particular, it has been reported that mitochondrial stress-dependent mtROS generation, could result in a dual dose-dependent response, namely: a harmful one, when the levels of the mtROS generated are high; and a protective one, associated with increased health and lifespan, when the levels of mtROS generated are low (Schulz et al., 2007). These observations lead to the formulation of the “mitohormesis” theory, in which a mild and sub-lethal mitochondrial stress activates a broad and complex cytosolic and nuclear response (Figure 1), able to promote long-lasting metabolic and biochemical changes, and to improve health and viability (Tapia, 2006; Yun and Finkel, 2014; Barcena et al., 2018). Key players in this scenario are mitochondria generated/released molecules, such as mtROS and mitokines, the role and nature of the latter still to be fully addressed (Durieux et al., 2011; Deng and Haynes, 2016; Zhang et al., 2018; Conte et al., 2019; Klaus and Ost, 2020), able to induce and regulate a complex signaling network between mitochondria, the nucleus (Quiros et al., 2016), other organelles, such as lysosomes (Ramachandran et al., 2019), and the cytosol (D’Amico et al., 2017), resulting in increased cell stress resistance that can improve health and extend lifespan. Of particular interest is the recent observation that early developmental transient redox stress in *Caenorhabditis elegans* could indeed modify the methylation state of the genome, and lead to increased stress resistance, improved redox homeostasis, and prolonged lifespan (Bazopoulou et al., 2019).

In addition, the activation of the mitohormetic response has a huge impact not only on the regulation of pro-survival pathways, such as the ones controlling mitochondrial dynamics and quality (Palikaras et al., 2015; Palmeira et al., 2019), but also on cell killing ones, and could result in the preservation rather than elimination of post-mitotic cells, such as neurons (Hekimi et al., 2016).

## Mitochondrial Dynamics in Aging Brain

**FIGURE 2:**
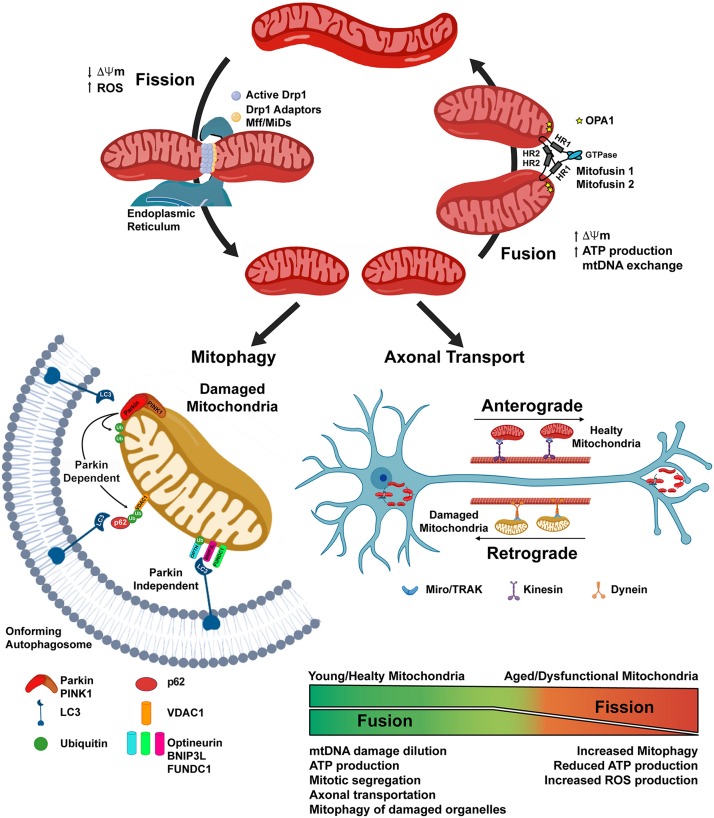
Mitochondrial dynamics depends on the balance of Fission and Fusion processes. Mitochondrial fission is initiated through the interaction of mitochondria with the Endoplasmic Reticulum and the actin cytoskeleton. The activation of the Dynamin related protein-1 (Drp1) allows its translocation to the mitochondrial outer membrane, where with the help of the mitochondrial fission factor (Mff) and the mitochondrial dynamics proteins (MiDs), constrains the organelle until it divides into two separate ones. Mitochondrial fusion is dependent on the action of Mitufusin-1 and -2, on the outer mitochondrial membrane (OMM), and of OPA1, on the inner mitochondrial membrane. Fission is not only a physiological event, i.e., allowing the organelles to be transported along axons or correctly segregated in daughter cells during mitosis, but could also be a consequence of mitochondrial damage, such as the loss of the mitochondrial membrane potential (ΔΨm), thus allowing the removal of damaged organelles through the mitophagy process. Parkin-dependent mitophagy requires the PINK1-dependent recruitment of the cytosolic Parkin on the OMM, where it could ubiquitinate mitochondrial resident protein, such as VDAC1. This protein signal is later recognized by the adaptor protein p62 which, through the interaction with LC3 on the membrane of the on-forming autophagosome, delivers the targeted mitochondria to the degradation. In the Parkin-independent mitophagy, the damaged organelle is recognized by LC3 through the interaction with other protein receptors, such as BNIP3L, FUNDC1, Optineurin, localized on the outer mitochondrial membrane.

Mitochondria are dynamic organelles that form a complex cellular network, which is constantly subjected to fission and fusion processes, and whose “healthy state” is maintained through mitophagy. Mitochondrial network modifications allow the cells: to relocate the organelles where more energy is required, a feature of importance in neurons with extensive axons and numerous dendrites (Mandal and Drerup, 2019); to guarantee the right number of mitochondria in each daughter cell after division (Mishra and Chan, 2014); and are also involved in the quality control of the organelle. Indeed, mitochondrial fusion allows mitochondria to increase OXPHOS and ATP production, to exchange mitochondrial DNA (mtDNA), and to dilute possible organelle’s damage (Sebastián et al., 2017). On the other hand, mitochondrial fission results into network fragmentation, leading to increased ROS production and signaling toward cell death by apoptosis, but also allow correct mitochondrial division during cell division, and the isolation of damaged organelles, which would then be degraded by mitophagy (Simula et al., 2017). The regulation of these processes is tightly regulated and relies on the action of several proteins. Fusion of the outer mitochondrial membrane (OMM) is mainly dependent on the GTPase Mitofusin 1 and 2 (Mfn-1 and -2) (Eura et al., 2003); whilst fusion of the inner mitochondrial membrane occurs thanks to the activity of the dynamin-related GTPase Optic-Athropy-1 (Opa-1) protein (Cipolat et al., 2004). Fission of the mitochondrial network occurs through the activity of the GTPase Dynamin-Related-Protein-1 (Drp1) (Oliver and Reddy, 2019; Qi et al., 2019). After an initial constriction of the OMM, due to the interaction with the endoplasmic reticulum and actin filaments, Drp-1 forms multimeric spirals around the OMM, which further constrict until they divide the membranes, using GTP hydrolysis to catalyze this process (Kraus and Ryan, 2017; van der Bliek et al., 2017) (Figure 2).

Mitochondrial dynamics are at the basis of healthy aging (Sharma et al., 2019) and their dysregulation, such as increased mitochondrial fission and reduced fusion are prominent features both in physiological aging and neurodegenerative conditions. Indeed, Drp1 hyperactivity has been reported for AD, PD, HD, Down syndrome, multiple sclerosis, and amyotrophic lateral sclerosis (ALS) (Oliver and Reddy, 2019). Moreover, fusion/fission imbalance has been proposed to cause impairment in memory and learning (Li et al., 2019; Yan et al., 2019), as mitochondrial network dynamics are pivotal in neurons, in order to localize the organelles in dendrites, axon, and synapses, located far away from the cell body. Mutations in the Drp1 and OPA1 proteins results in the lack of mitochondria on axon terminals, with consequent impairment of synaptic transmission, the main cause of cognitive deficits (Li et al., 2004).

The “health” of the mitochondrial network, relies on the action of a complex mitochondrial quality control program, involving different pathways that regulates: the elimination of improperly folded proteins, via the action of mitochondrial resident proteases and of a process termed OMM associated degradation (Pickles et al., 2018); the mitochondrial biogenesis, in which the coordination of nuclear and mitochondrial genome is crucial; the possible recovery and/or demise of damaged organelles, through the activation of the mitochondrial unfolded response (UPR^*mt*^) (Shpilka and Haynes, 2018), and mitophagy (Pickles et al., 2018). Mitophagy is a specialized form of autophagy, in which damaged mitochondria are engulfed into autophagosomes, which then fuse with lysosomes allowing the degradation of the cargo (Galluzzi et al., 2017; Rodolfo et al., 2017). Damaged mitochondria are flagged for recruitment to the autophagosome thanks to the activation of the PTEN-induced putative kinase 1 (PINK1) protein in the OMM, which in turn allow the recruitment of the ubiquitin ligase Parkin from cytosol. Parkin activity on the OMM proteins, allows their ubiquitination and recognition by different protein receptors (optineurin, p62, NBR1 and others) located in the forming autophagosome (Figure 1). Mitophagy is an active process that requires energy, thus when ATP levels decrease, as with age, the process does not occur at the same rate, and the accumulation of dysfunctional mitochondria in the neuronal soma could inhibit mitochondrial axonal transport, thus leading to neurodegenerative disease onset as well as to cognitive deficit (Kerr et al., 2017).

## From Bench to Bedside

In the last decade, the search toward the development of new therapeutic and pharmacological strategies, able to counteract the progression of neurodegenerative diseases and/or to ameliorate the life of the elder people, pointed to the identification of molecules able to regulate mitochondrial dynamics and/or general and specific autophagy mechanisms. Mitochondrial fission depends upon the activation of the Drp1 protein, so different drugs able to inhibit this process have been identified and tested both *in vitro* and *in vivo* (for a complete review see Oliver and Reddy, 2019), among them: Mdivi-1, inhibits excessive mitochondrial fission and promotes fusion, and is being considered for clinical trials for human diseases (Cassidy-Stone et al., 2008; Reddy et al., 2018); the selective peptide inhibitor P110, proved to be effective on a PD mouse model (Qi et al., 2013); Dynasore has been discovered as a specific inhibitor of Dynamin-1 and -2 as well as of Drp1 (Macia et al., 2006), but there are just few reports on its efficacy on mitochondria (Gao et al., 2013); the small molecule DDQ [diethyl (3,4-dihydroxyphenethylamino) (quinolin-4-yl) methylphosphonate)], proved to be able not only to reduce mitochondrial fission and to enhance fusion, but also to induce mitochondrial biogenesis, and a reduction of both beta-amyloid and Drp1 proteins in APPSwe/Ind cells (Kuruva et al., 2017). The obtained results suggested these compounds as promising candidates able to reduce excessive mitochondrial fission, as to ameliorate neuronal mitochondrial functionality as well as the cognitive deficit, in AD, PD, and HD patients. Nevertheless, there is still a lot to be done before anyone of this drugs could be authorized for the treatment in humans.

Macroautophagy and mitophagy are highly regulated mechanisms and, as of today, any of the possible interventions, specifically aimed at modulating them, are not available for use in humans. This is mostly due to the fact that drugs licensed for the use in humans and able to activate or inhibit autophagy (i.e., rapamycin, chloroquine, hydroxychloroquine, etc.) were not developed for this purpose, they show limited specificity toward the autophagic process and/or the target cell, and most of their targets have also an autophagy-independent function. In addition, there is a lack of suitable and precise biomarkers, to monitor the efficacy of the treatment, as well as of more physiological *in vivo* models of autophagy deficiencies (Galluzzi et al., 2017). In neurodegenerative diseases, showing defective autophagy as a common feature, the identification of the precise point at which autophagy fails and the development of drugs able to target specific types and/or steps of the autophagic process, are the major points on which being focused in the future (Scrivo et al., 2018). In particular, there is growing interest in the possibility of modifying the number of mitochondria, their dynamics, or the mitochondrial quality-control system, in pathological instances in which any of these were impaired or abnormal. As of today, there are no really effective strategies to do so (Georgakopoulos et al., 2017), and it should be taken in account that these processes are the result of a complex interplay between different cellular pathways, and the alteration of one of them could severely affect the others (Strappazzon and Cecconi, 2015; Strappazzon et al., 2015). In this scenario, the increasing knowledge about this interplay could be beneficial to the development of combined therapeutic approaches, aiming to different aspects such as, ROS formation targeting, mitochondrial biogenesis stimulation, mitochondrial dynamics modulation (Elfawy and Das, 2019). For example, development of new delivery systems able to enhance the antioxidant bioavailability or administration of a diet enriched in natural compounds able to stimulate mitophagy, could be coupled to unique mitochondrial proteins identified as specific targets for the newly developed drugs (Palikaras et al., 2017; Di Rita et al., 2018; Di Rita and Strappazzon, 2019). In this view, the future improvement of new technical approaches, such has optogenetic, could be of importance in the design and development of effective therapies (D’Acunzo et al., 2019).

Another important aspect to be taken in account, relies on the cellular heterogeneity of the central nervous system (CNS) and to cell specific response to a specific treatment, both in physiological and pathological conditions. We should foster a more complete picture of cell’s interactions, mitochondrial heterogeneity, and autophagy regulation in the CNS, in order to develop new compounds, capable of targeting autophagy and/or mitophagy in specific cell types or brain region. In this scenario, the recent development of new and better analytical methods, to study mitochondria in normal and diseased states, took advantage from the development of software, such as MitoMo (Zahedi et al., 2018), in which machine learning was used to analyze data obtained by different mitochondrial imaging approaches, thus allowing a deep analysis of mitochondrial features in any cell type. This kind of development would be more and more important when coupled to the development of methods, able to target mitochondria *in vivo*, for both imaging and isolation. As an example, the use of the *MitoTag* mice, highlighted that in the brain, not only cells are of different types, but also mitochondria showed cell-type-specific functional and molecular diversity, with hundreds of differentially regulated mitochondrial proteins, which could be used as cell-type-specific mitochondrial markers in healthy vs. diseased CNSs (Fecher et al., 2019).

Restoration or amelioration of mitochondrial functionality in pathological settings, is not the only field to be explored and implemented in the future, as deeper knowledge of the mechanisms underlying memory engravement, storage, and recall could open a new scenario to ameliorate the life of the elders. In this scenario, we could envision the development and improvement of technology, allowing the labeling and manipulation of specific cell ensembles, responsible for memory engrams, which have been shown to be sufficient for memory recall (Tonegawa et al., 2015; de Sousa et al., 2019), also in an animal model of neurodegeneration (Roy et al., 2016). Nowadays, a direct link between mitochondrial activity and memory formation remains quite elusive, even if it has been reported a crucial role in the cannabinoid-induced amnesia (Hebert-Chatelain et al., 2016) and the interesting observation that long-term potentiation in neurons requires a rapid burst of dendritic mitochondrial fission as well as an increase in the concentration of mitochondrial matrix Ca^2+^ (Divakaruni et al., 2018).

Of great interest is the recent report about the existence of a phenomenon termed “mitoengram,” in which transient stress results in enduring physio-chemical changes of the mitochondrial network and couple to key epigenetic modifications, in muscle stem cells (Cheikhi et al., 2019). Whether this role for mitochondria and mitochondrial dynamics in the balance between persistent and transient cellular memory could also happen in other cell types, such as neurons, as well as its potential exploitation as a therapeutic target, are a new research field worth to be investigated.

## Conclusion

The last decade gave us a bunch of knowledge about cellular processes at the basis of aging and its linked pathologies. Our understanding is growing day by day and the continuous development of new technologies allow us to imagine a wide field of therapeutic approaches to ameliorate the life of “aged people” as well as to counteract the onset and progression of neurodegenerative diseases. Nevertheless, a lot remains to be discovered and big efforts would be necessary in order to effectively transfer the obtained knowledge from bench to the bedside.

## Author Contributions

FM and CR wrote the manuscript. CR and FC revised the manuscript.

## Conflict of Interest

The authors declare that the research was conducted in the absence of any commercial or financial relationships that could be construed as a potential conflict of interest.
